# The cumulative live birth rate of recombinant follicle-stimulating hormone alfa verse urinary human follicle-stimulating hormone for ovarian stimulation in assisted reproductive technology cycles

**DOI:** 10.1186/s13048-022-01009-w

**Published:** 2022-06-21

**Authors:** Chunxia Yang, Naijun Dong, Feng Li, Yurong Ji, Yu Pan, Hong She

**Affiliations:** Reproductive Medicine Center, Northern Jiangsu People’s Hospital, Address: 98 Nantong West Road, Yangzhou, Jiangsu China

**Keywords:** Ovulation induction, Embryo transfer, Oocyte retrieval, Follicle stimulating hormone, Ovarian Hyperstimulation syndrome

## Abstract

**Background:**

Infertility remains a significant public health concern. An issue with controlled ovarian stimulation (COS) is the selection of an exogenous gonadotropin (Gn) regimen, which is mainly based on urinary follicle-stimulating hormone (uFSH), recombinant follicle-stimulating hormone alfa (rFSH-alfa), and human menopausal gonadotropin (HMG). In addition, most previous studies focused on the clinical pregnancy rates or live birth rates (LBR) per transfer cycle, but not on the cumulative live birth rate (CLBR) per started cycle. The CLBR, appears to be a more comprehensive and accurate universal measure of IVF treatment success. Therefore, this study aimed to compare the cumulative live birth rate (CLBR) between rFSH-alfa and uFSH regimens for ovarian stimulation.

**Methods:**

This retrospective cohort study included patients who underwent assisted reproductive technology (ART) with gonadotropin-releasing hormone (GnRH) agonist long protocol between March 2009 and December 2018. Patients were grouped according to the Gn regimen received (rFSH-alfa or uFSH). The main outcome was CLBR, which defined as the first live birth following the use of all fresh and frozen embryos derived from a single COS cycle.

**Results:**

A total of 1078 cycles were analyzed (314 with rFSH-alfa and 764 with uFSH). The rFSH-alfa group was characterized by a higher number of retrieved oocytes (13.3 vs. 11.0) and transferable embryos (5.0 vs. 4.0), a higher fresh embryo transfer rate (35.0% vs. 26.3%), and a higher multiple birth rate among the fresh embryo transfer cycles (8.2% vs. 2.5%) (*P* < 0.05). There were no differences in pregnancy rate (32.7% vs. 33.8%) and LBR (25.5% vs. 26.9%) per transfer cycle (*P* > 0.05). No significant difference was found in clinical outcomes among the frozen embryo transfer cycles (*P* > 0.05). The CLBR per started cycle in the rFSH-alfa group was higher than in the uFSH group (53.5% vs. 43.1%, *P* < 0.05). After adjustment, rFSH-alfa was independently associated with a higher CLBR (OR = 1.56; 95%CI = 1.18–2.05; *P* = 0.0018).

**Conclusions:**

rFSH-alfa and uFSH have similar pregnancy rates and LBR per transfer cycle, rFSH-alfa might achieve more transferrable blastocysts and higher CLBR per started cycle compared to uFSH.

**Supplementary Information:**

The online version contains supplementary material available at 10.1186/s13048-022-01009-w.

## Introduction

Infertility remains a significant public health concern, affecting about 15% of all couples of reproductive age [1], with a global prevalence of about 50–70 million couples [2]. Since the first in vitro fertilization (IVF) in 1978, assisted reproductive technology (ART) with controlled ovarian stimulation (COS) has become an effective infertility treatment. An issue with COS is about the selection of an exogenous gonadotropin (Gn) regimen, which is mainly based on urinary follicle-stimulating hormone (uFSH), recombinant follicle-stimulating hormone alfa (rFSH-alfa), and human menopausal gonadotropin (HMG) [3]. The European Society of Human Reproduction and Embryology (ESHRE) published a guideline on COS in 2020, with recommendations on Gn [4]. Still, the consensus is based on previous evidence concluding that there is no difference between recombinant and urinary FSH, which is based only on clinical pregnancy rates or live birth rates (LBR) per transfer cycle, but not on the cumulative live birth rate (CLBR) per started cycle [4]. Indeed, there have been many studies comparing clinical pregnancy or LBR per transfer cycle between rFSH-alfa and uFSH [5–8], but no data related to the CLBR are available.

Outcomes traditionally used to evaluate results of in vitro fertilization, and embryo transfer (IVF-ET) treatment include the number of embryos obtained per IVF cycle, clinical pregnancy rate and LBR per transfer cycle (fresh or frozen cycle, respectively) [9]. Nevertheless, with the widespread use of embryo cryo-resuscitation technology to increase the LBR, the above outcomes are not sufficient to evaluate the clinician’s concern regarding the length of the treatment and relieve the patient’s concern about the number of hospital visits [10]. Therefore, the CLBR, which is defined as the first live birth following the use of all fresh and frozen embryos derived from a single COS cycle, appears to be a more comprehensive and accurate universal measure of IVF treatment success [10–12] for both fresh embryo transfer and subsequent frozen-thawed embryo transfer (FET) [13]. Therefore, more and more investigators in recent years have begun to use the CLBR to evaluate the therapeutic effect of IVF, and the recognition of CLBR as the universal outcome and even a “gold standard” continues to expand [10, 14–16].

At present, the rFSH-alfa and uFSH are widely used for COS in China [17, 18]. Still, there is a lack of comprehensive evidence comparing the efficacy of COS regimens using these two drugs in China. In addition, despite CLBR recently becoming the gold standard for evaluating the efficacy of COS, there is no large-scale study comparing the CLBRs between rFSH-alfa and uFSH protocols [18].

Therefore, this study aimed to compare the clinical outcomes, particularly CLBR, between rFSH-alfa and uFSH regimens for COS in a real-world setting. The results could provide additional evidence for selecting the most optimal treatment for COS.

## Methods

### Study design and patients

This retrospective study included patients who underwent ART with COS at the Center for Reproductive Medicine of Subei People’s Hospital of Jiangsu Province from March 2009 to December 2018. STROBE guideline was followed in our preparation. This study was approved by the ethics committee of the Reproductive Medicine of Subei People’s Hospital of Jiangsu Province. Due to the retrospective nature, the need for informed consent was waived by the committee.

The inclusion criteria were 1) underwent gonadotropin-releasing hormone (GnRH) agonist long protocol, including short-acting GnRH agonist long protocol and long-acting GnRH agonist long protocol, 2) received rFSH-alfa or uFSH for COS, 3) ≤2 previous stimulation cycles, and 4) antral follicle count (AFC) ≥5.

### COS protocols

The depot GnRH agonist long protocol was used for COS. For the long-acting long protocol, a long-acting agonist analog was given on day 2–3 of menstruation for downregulation, with blood tests for FSH, luteinizing hormone (LH), and estradiol (E2) and a vaginal ultrasound 28–35 days after downregulation to check the status of downregulation. For the short-acting GnRH agonist long protocol, a short-acting agonist analog was given on day 21 of the previous menstrual cycle or 7 days after ovulation of the previous menstrual cycle. Blood was drawn for FSH, LH, and E2, and a vaginal ultrasound was performed on days 2–3 after the return of menstruation to check the status of downregulation. If pituitary down-regulation was confirmed, exogenous Gn was given to initiate COS. The FSH received by patients during the study period included rFSH-alfa (Gonal-F®, Merck Serono, Switzerland) or uFSH (Lizhu Pharmaceutical Trading Co., Zhuhai, China). The Gn initiation dose was 100–225 IU/d for patients with normal ovarian reserve function and 225–300 IU/d for patients with low reserve function. The drug dose was adjusted according to the growth rate of the follicles and hormone levels. During stimulation, the ovarian response was monitored by measuring serum E2, progesterone (P4), and LH and using serial transvaginal ultrasonographic examinations. Recombinant human choriogonadotropin (hCG) was administered until at least one follicle was observed with a diameter of ≥19 mm or two with diameters of ≥18 mm.

Oocyte retrieval occurred 36–40 h after hCG injection (trigger). IVF or ICSI were performed 4–6 h after in vitro culture of the oocyte. ICSI was performed in cases of males with severe oligospermia or azoospermia requiring testicular or epididymal puncture, previous IVF fertilization failure, fertilization rate < 20%, or normal fertilization rate < 30%; otherwise, IVF was performed. A pronucleus was observed 18–20 h after insemination. The Vitrolife serial sequential culture medium was used for embryo culture.

### Embryo transfer, luteal support, and follow-up

For patients with high risk of ovarian hyperstimulation syndrome (OHSS), substandard endometrial thickness or morphology, or moderate to severe hydrosalpinx, frozen embryo transfer is recommended; otherwise, fresh embryo transfer will be utilized. Fresh embryo transfer was performed 48 or 72 h after oocyte retrieval using a Cook soft catheter, while FET was performed on the day of thawing or day 1 post-thawing. The greatest endometrial thickness in the uterus’s longitudinal plane in transvaginal ultrasound was measured from one endometrial-myometrium interphase to the corresponding interphase. The measurement recorded on the day of hCG administration was used as the referral value in this study. Pregnancy was first assessed by a positive hCG test 2 weeks after oocyte retrieval and confirmed by ultrasound visualization of an intrauterine gestational sac with an embryo with heartbeats at 6 weeks gestational age.

### Data collection and definition

Patients’ information through the electronic medical record (EMR) were retrospectively collected, including baseline data (age, body mass index [BMI], duration of infertility, primary causes of infertility, the previous number of IVF/ICSI cycles, and baseline endocrine information), COS treatment and laboratory embryo culture (Gn total dose, 2PN number, IVF/ICSI, number of transferable embryos, number of high-quality embryos, and number of embryo transfer cycles). Clinical outcomes, including CLBR per started cycle, LBR per transfer cycle, pregnancy rate per transfer cycle, abortion rate, and multiple birth rate, were calculated.

High-quality embryos were defined as grade I and II embryos, based on the size, regularity, refractive index of the cytoplasm, and the number of fragments. Grade I embryos: uniform cell size, regular shape, complete zona pellucida, uniform and clear cytoplasm without granular phenomenon, and fragmentation between 0 and 5%. Grade II embryos: slightly uneven cell size, slightly irregular shape, the cytoplasm may be granular, and fragmentation between 6 and 20%. Grade III embryos: obviously uneven cell size, obviously irregular shape, the cytoplasm may be granular, and fragmentation between 21 and 50%. Grade IV embryos: severely uneven cell size, severely granular cytoplasm, and fragmentation > 50%.

Live birth was defined as the final birth of at least one living child born after 28 weeks of gestation. CLBR was defined as the first live birth following the use of all fresh and frozen embryos derived from a single COS cycle. In cases when all embryos were used up in one embryo retrieval cycle, or if a live birth was not achieved for more than 2 years after ART started, we considered that this embryo retrieval cycle finally did not achieve a live birth. LBR per transfer cycle was calculated as the number of live birth cycles divided by number of transfer cycles. Miscarriage rate was calculated as the number of miscarriage cycles divided by number of clinical pregnancy cycles. Multiple birth rate was calculated as the number of multiple live birth cycles divided by transfer cycles. Clinical pregnancy was defined as a transvaginal ultrasound 4 weeks after transplantation or 2 weeks after a positive hCG blood test indicating a gestational sac in or outside the uterine cavity. Clinical pregnancy rate per transfer cycle was calculated as the number of clinical pregnancies divided by number of transfer cycles.

### Statistical analysis

All statistical analyses were performed using Empower Stats (www.empowerstats.com, X&Y solutions, Inc. Boston, MA, USA) and R 3.6.1 (http://www.r-project.org). The continuous variables were tested for normality using the Kolmogorov-Smirnov test. The data complying with a normal distribution were presented as means ± standard deviations; otherwise, they were expressed as medians (25th–75th percentile). Categorical variables were expressed as the number of cases (percentage). The data between the two groups were compared and analyzed using non-parametric tests or the chi-square test, as appropriate. A logistic regression model was used to assess the relationship between FSH regimens and CLBR after adjusting for age, BMI, baseline FSH, and AFC. We selected confounders based on the associations reported with the outcomes of interest [6, 14–16]. A 1:2 propensity score matching (PSM) was also used to balance factors, including age, BMI, basic FSH, and AFC. Two-tailed *P* < 0.05 was considered statistically significant.

## Results

### General characteristics of the patients

This study included 1078 ART cycles with the GnRH agonist long protocol. Of them, 314 cycles were in the rFSH-alfa group, and 764 cycles were in the uFSH group, respectively. The patients in the uFSH group were 29.4 ± 3.8 years of age and had a mean BMI of 22.1 ± 3.6 kg/m^2^, while the patients in the rFSH-alfa group were 29.1 ± 3.6 years of age and had a mean BMI of 22.1 ± 3.6 kg/m^2^. The two groups were comparable for all baseline characteristics except AFC, which was slightly higher in the rFSH-alfa group (13.4 ± 4.4 vs. 12.3 ± 4.1, *P* < 0.001). The characteristics of the patients are shown in Table 1.

**Table 1:** Characteristics of the patients

Variables	uFSH (*n* = 764)	rFSH-alfa (*n* = 314)	P
Age (years)	29.4 ± 3.8	29.1 ± 3.6	0.310
BMI (kg/m^2^)	22.4 ± 3.2	22.1 ± 3.6	0.206
Duration of infertility (years)	3.0 (2.0–5.0)	3.0 (2.0–4.0)	0.331
Basic AMH (ng/ml)	3.14 (1.95–5.11)	3.63 (2.57–5.41)	0.147
AFC	12.3 ± 4.1	13.4 ± 4.4	< 0.001
Basic FSH (IU/L)	6.21 ± 1.72	6.38 ± 1.89	0.144
Infertility type			0.837
Primary	465 (60.9%)	189 (60.2%)	
Secondary	299 (39.1%)	125 (39.8%)	
Primary cause of infertility			0.174
Tubal	456 (76.0%)	182 (82.4%)	
Endometriosis	43 (7.2%)	16 (7.2%)	
PCOS	77 (12.8%)	15 (6.8%)	
Male	7 (1.2%)	3 (1.4%)	
Other causes	17 (2.8%)	5 (2.3%)	
Previous number of IVF/ICSI cycles			0.059
0	659 (86.3%)	284 (90.5%)	
1	105 (13.7%)	30 (9.6%)	

*BMI* body mass index; *AMH* anti–Müllerian hormone; *AFC* antral follicle count; *FSH* follicle-stimulating hormone; *PCOS* polycystic ovary syndrome; *IVF* in vitro fertilization; *ICSI* intracytoplasmic sperm injection

### COS treatment and embryo culture

The overall Gn dose needed to achieve ovulation was significantly lower with rFSH-alfa than with uFSH (1912.3 ± 625.1 vs. 2334.5 ± 687.3 IU, *P* < 0.001). As shown in Table 2, the mean number of oocytes retrieved (13.0 vs. 10.0), 2PN oocytes (7.0 vs. 5.0), and transferable embryos (5.0 vs. 4.0) in the rFSH-alfa group were higher than in the uFSH group (all *P* < 0.05).

**Table 2:** COS treatment and embryo culture

Variables	uFSH (*n* = 764)	rFSH-alfa (*n* = 314)	P
Gn total dose (IU)	2334.5 ± 687.3	1912.3 ± 625.1	< 0.001
Oocytes retrieved	10.0 (6.8–14.0)	13.0 (8.0–17.0)	< 0.001
2PN oocytes	5.0 (3.0–8.0)	7.0 (4.0–11.0)	< 0.001
Transferrable embryos	4.0 (2.0–6.0)	5.0 (3.0–8.0)	< 0.001
High-quality embryos	3.0 (1.0–5.0)	4.0 (1.0–6.0)	< 0.001
OHSS	36 (4.7%)	22 (7.0%)	0.129
IVF/ICSI			0.014
ICSI	118 (15.4%)	68 (21.7%)	
IVF	646 (84.6%)	246 (78.3%)	
Number of embryo transfer cycles			< 0.001
0	141 (18.5%)	32 (10.2%)	
1	408 (53.4%)	154 (49.0%)	
2	152 (19.9%)	90 (28.7%)	
3	57 (7.5%)	25 (8.0%)	
4	6 (0.8%)	10 (3.2%)	
5	0 (0.0%)	3 (1.0%)	

*COS* controlled ovarian stimulation; *OHSS* ovarian hyperstimulation syndrome; *IVF* in vitro fertilization; *ICSI* intracytoplasmic sperm injection; *FSH* follicle-stimulating hormone

### Clinical outcomes in the uFSH group and rFSH-alfa group

The clinical outcomes were analyzed and compared between the two groups and between the fresh and frozen embryo transfer subgroups in each group, as shown in Table 3. The CBLR was higher with rFSH-alfa than with uFSH (53.5% vs. 43.1%, *P* = 0.002). The fresh embryo transfer rate (35.0% vs. 26.3%) and the multiple birth rate in the fresh embryo transfer cycles (8.2% vs. 2.5%) in the rFSH-alfa group were higher than in the uFSH group (all *P* < 0.05). In the fresh transfer subgroup, there were no differences in the pregnancy rate (32.7% vs. 33.8%), the abortion rate (11.1% vs. 11.8%), and LBR (77.8% vs. 79.4%) among the groups in the fresh embryo transfer cycles (all *P* > 0.05). For the FET cycles, there were no differences in the clinical pregnancy rate (47.7% vs. 48.7%), abortion rate (13.6% vs. 16.3%), LBR (40.1% vs. 39.1%), and multiple birth rate (7.9% vs. 7.5%) between the two groups (all P > 0.05).

**Table 3:** Clinical outcomes

Varibales	uFSH (*n* = 764)	rFSH-alfa (*n* = 314)	P
CLBR	329 (43.1%)	168 (53.5%)	0.002
Time to first live birth (days)	404.0 (357.0–496.0)	392.0 (356.0–452.8)	0.471
Fresh embryo transfer, n	201 (26.3%)	110 (35.0%)	0.005
Pregnancy, n/N (%)	68/201 (33.8%)	36/110 (32.7%)	0.844
Abortion	8/68 (11.8%)	4/36 (11.1%)	0.921
Live birth	54/201 (26.9%)	28/110 (25.5%)	0.787
Multiple birth	5/201 (2.5%)	9/110 (8.2%)	0.021
Frozen embryo transfer cycles	706	354	
Pregnancy, n/N (%)	344/706 (48.7%)	169/354 (47.7%)	0.762
Abortion	56/344 (16.3%)	23/169 (13.6%)	0.431
Live birth	276/706 (39.1%)	142/354 (40.1%)	0.749
Multiple birth	53/706 (7.5%)	28/354 (7.9%)	0.816

*CLBR* Cumulative live birth rate; *FSH* follicle-stimulating hormone

### Univariable and multivariable analysis for CLBR before and after PSM

The univariable analyses showed that rFSH-alfa (OR = 1.52, 95%CI: 1.17–1.98, *P* = 0.0018), age (OR = 0.94, 95%CI: 0.91–0.97, *P* < 0.0001), and AFC (OR = 1.04, 95%CI: 1.01–1.07, *P* = 0.0031) were associated with higher CLBR in the total study population. The multivariable analysis showed that rFSH-alfa (OR = 1.56, 95%CI: 1.18–2.05, *P* = 0.018) and age (OR = 0.94, 95%CI: 0.91–0.98, *P* = 0.0013) were independently associated with a higher CLBR (Table 4). After PSM, it was also found that the CLBR in the rFSH-alfa group was higher than in the uFSH group (53.5% vs. 43.1%, *P* = 0.0004) (Table S1).

**Table 4:** Univariable and multivariable analysis for CLBR

Variables	Univariable		Multivariable	
	OR (95%CI)	P	OR (95%CI)	P
Group				
uFSH	Ref		Ref	
rFSH-alfa	1.52 (1.17, 1.98)	0.0018	1.56 (1.18, 2.05)	0.0018
Age	0.94 (0.91, 0.97)	< 0.0001	0.94 (0.91, 0.98)	0.0013
BMI	1.01 (0.97, 1.05)	0.6825	1.01 (0.97, 1.05)	0.7216
Basic FSH	0.93 (0.87, 1.00)	0.0414	0.95 (0.88, 1.03)	0.2094
AFC	1.04 (1.01, 1.07)	0.0031	1.02 (0.99, 1.06)	0.1718

*CLBR* Cumulative live birth rate; *FSH* follicle-stimulating hormone; *BMI* body mass index; *AFC* antral follicle count; *OR* odds ratio; *CI* confidence interval

## Discussion

This study aimed to compare the clinical outcomes, particularly CLBR, between rFSH-alfa and uFSH regimens for ovarian stimulation in a real-world setting. Our main finding is that after adjusting potential confounders (age, BMI, and AFC), rFSH-alfa was independently associated with a higher CLBR compared with uFSH (OR = 1.56, 95%CI: 1.18–2.05, *P* = 0.001). To the best of our knowledge, this is the first real-world study that used CLBR as a main outcome on a relatively large sample size and reported significant differences between rFSH-alfa and uFSH.

In addition, we found that the mean number of oocytes retrieved (13.3 vs. 11.0), 2PN oocytes (7.0 vs. 5.0), and transferable embryos (5.0 vs. 4.0) in the rFSH-alfa group were higher than in the uFSH group. There were no differences in the incidence of OHSS (7% vs. 4.7%), pregnancy rate (32.7% vs. 33.8%), abortion rate (11.1% vs. 11.8%), and LBR (77.8% vs. 79.4%) among the groups both in the fresh and frozen embryo transfer cycles.

COS is an important part of assisted reproduction, and FSH is commonly used clinically to enhance recruitment and development of ovarian follicles. Ongoing issues with uFSH derived from urine donations include batch-to-batch variability and a finite donor supply [19]. Indeed, batch-to-batch variability can lead to imprecise dosage [20, 21], and the precise dosage of FSH is critical in COS [4]. Hazard issues with urinary products have also been suggested [21], including impurities [22]. The development of rFSH-alfa has mostly solved related problems. Previous studies have already reported that the pregnancy rates were higher for rFSH but failed to demonstrate differences in LBR [5–8]. Therefore, many physicians generally consider that for normal responders, a good single transfer cycle LBR can be obtained regardless of the Gn selected and that the selection of Gn is not that important. However, our study found that rFSH-alfa group showed higher oocyte retrieval with fewer FET cycle cancellations (10.0% in the rFSH-alfa group and 18.5% in the uFSH group) in young responders with good responses. Therefore, even in young patients with good responses, rFSH-alfa for COS may bring clear clinical benefits, and the flexible dose adjustment characteristics of rFSH-alfa should be considered to allow for a more individualized Gn initiation and dose adjustment. In our study, the LBR per transfer cycle also showed no difference between rFSH-alfa and uFSH, but the CLBR was higher in the rFSH-alfa group than in the uFSH group, together with the number of retrieved oocytes (13.3 vs. 11.0) and the number of mature oocytes (7.6 vs. 6.0). Those findings are in line with a large real-world study, reporting that the CLBR of a single ovulation induction cycle increases significantly with the number of obtained oocytes [23] and confirming that rFSH-alfa might be beneficial for the clinical outcomes of COS for ART.

rFSH-alfa is produced by inserting the FSH DNA into a Chinese hamster ovary cell line and has no detectable LH activity and very high specific bioactivity for FSH (> 10,000 IU/mg protein) [24, 25]. Highly purified human urinary Gn products are extracted from postmenopausal urine, and for each 75 IU of FSH, the LH content varies from < 1 to 75 IU, and the LH might influence the outcomes of COS or at least complicate the precise control of COS [24, 26]. FSH possesses different isoforms (more or less acidic, glycosylated, and sialylated isoforms), and the extraction conditions of FSH (e.g., pH) from the urine can affect the isoforms being present in the resulting uFSH preparations since the isoforms have different subunit assembly, intracellular sorting, metabolic clearance, and regulation of potency at the cell level [27]. LH activity promotes folliculogenesis in LH deficiency patients [28, 29], but how to precisely dose uFSH to optimize the outcomes of COS when it is unknown which LH content will be found in the next uFSH vial is a major issue. This variability potentially leads to variation in clinical effectiveness, caused not only by the normal problems associated with isolation and purification of hormones from the urine of postmenopausal women but also by the evidence that the bioactive content of uFSH varies considerably from batch to batch [26]. Studies that compared uFSH with rFSH-alfa noted increased ovarian recruitment of follicles in the rFSH-alfa group [30], more efficient ovarian response, and better quality of oocytes [31]. In our study, we also noted that the fresh embryo transfer rate (35.0% vs. 26.3% in the rFSH-alfa group were higher than in the uFSH group (*P* < 0.05).

Another recent large (> 28,000 women) real-world study demonstrated significantly higher rates of CLBR, cumulative ongoing pregnancy, and cumulative clinical pregnancy with r-hFSH-alfa compared to human menopausal gonadotropin [32]. The relatively large sample size using PSM in our study allowed us to statistically validate previous trends, in particular, that the overall FSH dose needed to achieve ovulation is significantly lower for rFSH-alfa [5, 33], and there is no evidence of a difference in the OHSS rate [34] noted by other studies.

This study has certain limitations. First, there is a risk of selection bias due to the retrospective nature of the study. In order to reduce this effect, we used multivariable analyses and PSM to ensure higher reliability of the obtained outcome. Second, we did not collect the medical costs of two groups in the study. Future studies are warranted to provide more comprehensive evidence for clinical decision-making. Third, we only compared the CLBR between rFSH-alfa and uFSH in patients received agonist long protocol. The different FSH in antagonist protocol will need to be studied in the future. Finally, our study is single-center research. Despite the large sample size, it might not represent other regions or nationalities.

## Conclusions

In conclusion, this study reports that despite rFSH-alfa and uFSH having similar pregnancy rates and LBR in both fresh embryo transfer cycle and FET cycle, rFSH-alfa achieved more transferrable blastocysts and higher CLBR compared with uFSH. Those results contribute to the discussion on the use of exogenous Gn regimens in COS and might help in optimizing COS strategies for clinical practice.

## Supplementary Information

**Additional file 1.**

## Data Availability

The datasets used and/or analysed during the current study are available from the corresponding author on reasonable request.

## Abbreviations

IVF: In vitro fertilization
ART: Assisted reproductive technology
COS: Controlled ovarian stimulation
Gn: Gonadotropin
uFSH: Urinary follicle-stimulating hormone
rFSH-alfa: Recombinant follicle-stimulating hormone alfa
HMG: Human menopausal gonadotropin
LBR: Live birth rates
CLBR: Cumulative live birth rate
FET: Frozen-thawed embryo transfer
AFC: Antral follicle count
LH: Luteinizing hormone
