# Supplementary material for: The cumulative live birth rate of recombinant follicle-stimulating hormone alfa verse urinary human follicle-stimulating hormone for ovarian stimulation in assisted reproductive technology cycles
Source: J Ovarian Res. 2022 Jun 21;15:74. doi: 10.1186/s13048-022-01009-w (PMC9210702; doi:10.1186/s13048-022-01009-w)
Supplement: Supplementary file 1 — Additional file 1. [file 13048_2022_1009_MOESM1_ESM.docx]

**Supplementary materials**

**Table S1.** CLBR in the uFSH group and rFSH-alfa group after PSM

| Variables | uFSH  (N=550) | rFSH-alfa  (N=275) | P |
| --- | --- | --- | --- |
| Age (years) | 29.4 ± 3.8 | 29.2 ± 3.6 | 0.5080 |
| BMI (kg/m^2^) | 22.2 ± 3.0 | 22.1 ± 3.3 | 0.8417 |
| AFC | 12.6 ± 4.1 | 12.9 ± 3.8 | 0.2818 |
| Baseline FSH (IU/L) | 6.21 ± 1.73 | 6.35 ± 1.81 | 0.3045 |
| CLBR | 227 (41.3%) | 150 (54.5%) | 0.0004 |

CLBR: Cumulative live birth rate; BMI: body mass index; AFC: antral follicle count; FSH: follicle-stimulating hormone; PSM: propensity score matching.
